# The methylome in females with adolescent Conduct Disorder: Neural pathomechanisms and environmental risk factors

**DOI:** 10.1371/journal.pone.0261691

**Published:** 2022-01-28

**Authors:** Andreas G. Chiocchetti, Afsheen Yousaf, Regina Waltes, Anka Bernhard, Anne Martinelli, Katharina Ackermann, Denise Haslinger, Björn Rotter, Nico Krezdorn, Kerstin Konrad, Gregor Kohls, Agnes Vetro, Amaia Hervas, Aranzazu Fernández-Rivas, Christine M. Freitag

**Affiliations:** 1 Department of Child and Adolescent Psychiatry, Psychosomatics and Psychotherapy, Goethe University Frankfurt, Frankfurt am Main, Germany; 2 GenXPro GmbH, Frankfurt am Main, Germany; 3 Department of Child and Adolescent Psychiatry, Psychosomatics and Psychotherapy, Child Neuropsychology Section, University Hospital, RWTH Aachen, Aachen, Germany; 4 Molecular Neuroscience and Neuroimaging, Institute of Neuroscience and Medicine (INM-11) JARA BRAIN Institute II, Research Center Juelich, Juelich, Germany; 5 Department of Pediatrics and Pediatrics Health Center, Child and Adolescent Psychiatry, Szeged, Szeged University, Szeged, Hungary; 6 Child and Adolescent Mental Health Service, Hospital Universitario Mutua de Terrassa, Barcelona, Spain; 7 Psychiatric Service, Basurto University Hospital, Osakidetza, Bilbao, Spain; Harvard Medical School, UNITED STATES

## Abstract

Conduct Disorder (CD) is an impairing psychiatric disorder of childhood and adolescence characterized by aggressive and dissocial behavior. Environmental factors such as maternal smoking during pregnancy, socio-economic status, trauma, or early life stress are associated with CD. Although the number of females with CD is rising in Western societies, CD is under-researched in female cohorts. We aimed at exploring the epigenetic signature of females with CD and its relation to psychosocial and environmental risk factors. We performed HpaII sensitive genome-wide methylation sequencing of 49 CD girls and 50 matched typically developing controls and linear regression models to identify differentially methylated CpG loci (tags) and regions. Significant tags and regions were mapped to the respective genes and tested for enrichment in pathways and brain developmental processes. Finally, epigenetic signatures were tested as mediators for CD-associated risk factors. We identified a 12% increased methylation 5’ of the neurite modulator *SLITRK5* (*FDR* = 0.0046) in cases within a glucocorticoid receptor binding site. Functionally, methylation positively correlated with gene expression in lymphoblastoid cell lines. At systems-level, genes (uncorr. *P* < 0.01) were associated with development of neurons, neurite outgrowth or neuronal developmental processes. At gene expression level, the associated gene-networks are activated perinatally and during early childhood in neocortical regions, thalamus and striatum, and expressed in amygdala and hippocampus. Specifically, the epigenetic signatures of the gene network activated in the thalamus during early childhood correlated with the effect of parental education on CD status possibly mediating its protective effect. The differential methylation patterns identified in females with CD are likely to affect genes that are expressed in brain regions previously indicated in CD. We provide suggestive evidence that protective effects are likely mediated by epigenetic mechanisms impairing specific brain developmental networks and therefore exerting a long-term effect on neural functions in CD. Our results are exploratory and thus, further replication is needed.

## Introduction

Conduct Disorder [1] is defined by symptoms of aggression towards people and animals, destruction of property, deceitfulness or theft, and violation of rules, with a childhood-limited, a childhood-persistent, and an adolescent-onset subtype [2–4]. In Europe, the prevalence of CD has been estimated to 1–3% in girls and 2–5% in boys, with rates increasing during puberty [5]. Especially females are most often diagnosed by the adolescent-onset, and presumably also by the adolescent-limited, subtype of CD [4]. In addition to genetic risk factors, twin and family studies have indicated a clear role of shared and non-shared environmental risk factors in the etiology of CD [6].

Among the best replicated environmental risk factors for CD, maternal smoking during pregnancy has been associated with increased risk for CD in a meta-analysis [7]. In addition, a number of psychosocial risk factors increase CD risk. These include low socio-economic status [8], adverse parenting, such as lack of parental warmth and early maltreatment, as well as history of trauma exposure, including but not limited to childhood maltreatment. Also, school and peer related problems have been observed more often in individuals with CD compared to typically developing peers [5, 9–13]. Some of these environmental factors may act at the epigenetic level and lead to altered gene expression, ultimately influencing long-term neural and brain development [14].

DNA methylation is an epigenetic marker dynamically regulating gene expression during development but also in mature cells including neurons. While this process is naturally occurring during the development of persisting cellular phenotypes, environmentally induced changes might also have detrimental effects, for example on synaptic function [15]. For instance, intrauterine exposure of human fetuses to maternal smoking was associated with an altered methylation of the G-protein coding gene *GNA15* and the Flavoprotein pseudogene *SDHAP3* in the dorsolateral prefrontal cortex. The authors also reported a significantly reduced number of neurons in this region [16].

Only few human studies have tested genome wide methylation patterns as mediators of environmental risks: In individuals with a history of trauma exposure, an altered cortisol stress response was mediated by a changed methylation of the gene *KITLG* (KIT tyrosin kinase ligand) [17]. In other studies, early life stress was associated with a hyper-methylation of the serotonin transporter *5HTT* gene [18] and of the *NR3C1* gene (coding for the glucocorticoid receptor) [19], which could by confirmed in a target analysis [20].

Studies focusing on conduct disorder or CD-associated behavior are rare: One of the largest studies so far reported seven loci to be differentially methylated (*FDR* < 0.05) comparing individuals with early-onset conduct problems (N = 174) to those with low conduct problems (N = 86). Three of these loci were adjacent to the genes *MGLL* (Monoglyceride Lipase), *TTBK* (Tau Tubulin Kinase) *GCET2* (Germinal Center-Associated Signaling And Motility Protein), [20]. In a study including 12 male healthy controls and 8 males with childhood chronic physical aggression (CPA) a suggestively significant differential methylation of promoters of neurotransmission-associated genes (e.g. *GRM4*, *AVPR1A*, *5HTR1D*, *SLC6A3*were reported [21, 22]. In a population-based twin study (n = 2,029) aggressive behavior (ASEBA Adult Self-Report) was significantly (*FDR* < 0.2) associated with two genes (*TRSP1* and *PARD6G*) and with neurotransmission at systems-level. However, based on the levels of significance reported, the findings need to pe replicates. Currently, no female specific effects were investigated [23].

In summary, there is evidence that aggression or associated phenotypic measures are related to differential methylation. However, no study has investigated methylation signatures in females with CD, and most studies only included autosomal regions in the analysis. To overcome this limitation, we investigated methylation in a female specific cohort of CD. In addition, by including only individuals with diploid X-chromosomes, we can include and analyze the X-chromosome as autosomes.

Finally, methylation is interacting with genetic variation (e.g. [24]), where common variants do explain ~ 19% of the physiological variance of methylation [25]. Thus, gene x methylation interaction (e.g. methylation might silence a risk allele) may explain variation in risk allele penetrance. Furthermore, sex-specific heritability of the methylation has been shown [25], suggesting that methylation signatures of male aggression might not be translatable to female aggression or CD. Studying males and females individually is becoming more important since only recently sex differences in CD have been identified at the neuronal level [26].

To our knowledge, only one genome-wide epigenetic study (EWAS) investigated the methylation signature of women with a history of chronic physical aggression (CPA N = 5, TD controls N = 14) and reported 404 differentially methylated regions (*FDR* < 0.05) in females with CPA, showing little overlap with the previously published male signatures, encouraging our approach to specifically investigate female specific signatures [21].

Considering these findings and the fact that female aggressive behavior and more specifically CD is generally under-researched, we aimed here to explore the epigenome of females with CD and its relation to environmental risks. Therefore, we first compared the DNA methylation pattern of late and post-pubertal female adolescents with CD to puberty matched female TDC without CD. Second, we explored if the differentially methylated genes identified were involved in specific biological and brain developmental processes at systems level. Finally, the identified differentially methylated loci were investigated as possible mediating mechanisms for the CD associated biological and psychosocial risk factors.

## Methods and materials

### Ethical considerations

All participants and/or their caregivers provided written informed consent prior to study enrollment. The study was positively reviewed by the respective local ethical committees (Frankfurt EK Universitätsklinikum No: 445/13; Aachen: EK der medizinischen Fakultät No: EK027-14; Barcelona Comite etico de invstigation clinica No: 12/14; Bilbao Comite etico de investigation clinica No: 2015-09-16 and 2016-07-20, Szeged Országos Tisztiföorvosi Hivatal, CRS/039/00392-3/2014).

### Participants

Study participants were recruited within the FemNAT-CD consortium within 2014–2018 across five European sites (Table 1, www.femnat-cd.eu [27]). After quality control (see below) N = 50 female adolescents with CD and N = 50 TD-control females matched by age, pubertal status according to the Pubertal Developmental Scale (PDS [28]), days between sample collection and DNA purification as well as DNA quality (260/280 ratio; Table 1, S1 File). CD was diagnosed according to DSM-5 [1] using a semi-structured interview with a parent/caretaker and the child (K-SADS-PL [29] with adjusted DSM-5 criteria). IQ was estimated by subtests of the Wechsler Intelligence Test or the Wechsler Abbreviated Scale of Intelligence [30–32] (further details see S1 File).

**Table 1:** Summary descriptive table by group.

	TD controls	CD-cases	p overall
	N = 50	N = 49	
Recruiting center			0.164 ^a^
Frankfurt (DE)	24 (48.0%)	13 (26.5%)	
Aachen (DE)	9 (18.0%)	17 (34.7%)	
Barcelona (ES)	5 (10.0%)	8 (16.3%)	
Bilbao (ES)	6 (12.0%)	6 (12.2%)	
Szeged (HU)	6 (12.0%)	5 (10.2%)	
Age (years)	16.1 (1.60)	15.8 (1.49)	0.404 ^a^
Number of CD symptoms	0.00 (0.00)	4.76 (2.50)	<0.001 ^b^
Pubertal Status			0.170 ^a^
Late pubertal	35 (70.0%)	41 (83.7%)	
Post pubertal	15 (30.0%)	8 (16.3%)	
Cigarettes per day	0.52 (2.08)	6.14 (6.57)	<0.001 ^b^
Contraceptives			0.025 ^a^
no	40 (80.0%)	28 (57.1%)	
yes	10 (20.0%)	21 (42.9%)	
History of int. disorder ^c^			<0.001 ^a^
no	45 (90.0%)	20 (40.8%)	
yes	5 (10.0%)	29 (59.2%)	
Current medication			0.001 ^a^
no	47 (94.0%)	32 (65.3%)	
yes	3 (6.00%)	17 (34.7%)	
Maternal smoking during pregnancy			0.071 ^a^
no	39 (81.2%)	24 (61.5%)	
yes	9 (18.8%)	15 (38.5%)	
Maternal exp. of aggression during pregnancy			0.001 ^a^
no	47 (97.9%)	30 (71.4%)	
yes	1 (2.08%)	12 (28.6%)	
Difficult family situation score (FamScore)	0.20 (0.49)	0.76 (0.80)	<0.001 ^b^
Parental Education (ISCED; EduPar)	8.11 (2.66)	5.94 (2.62)	<0.001 ^b^
Number of experienced traumata	0.86 (1.11)	2.16 (1.83)	<0.001 ^b^
DNA quality (260/280) ratio	1.84 (0.02)	1.84 (0.02)	0.748 ^b^
Time to DNA purification (days)	4.34 (3.01)	4.02 (3.38)	0.620 ^b^
Total reads per sample after cleaning	11,017,072 (5505663)	9,475,335 (4256539)	0.122 ^b^
Population Structure ^d^	N = 49	N = 47	
PC-1	0.00 (0.02)	0.00 (0.02	0.575 ^b^
PC-2	-0.01 (0.01)	0.00 (0.02	0.010 ^b^
PC-3	0.00 (0.01)	0.00 (0.03)	0.667 ^b^
PC-4	0.00 (0.02)	0.00 (0.02)	0.828 ^b^

a) Chi^2^ test

b) ANOVA

c) Internalizing disorder including depression, disruptive mood dysregulation disorder, anxiety or obsessive-compulsive disorder

d) PC: Principal component of common genetic variation; data was accessible for 96 individuals

#### Exclusion criteria

IQ <70, known monogenic disorder, any chronic or acute neurological disorder or history of epilepsy, traumatic brain injury, schizophrenia, current mania, bipolar disorder (BD) or autism spectrum disorder (ASD). TD controls were further excluded if fulfilling current or lifetime diagnostic criteria for CD, oppositional-defiant disorder (ODD), attention-deficit/hyperactivity disorder (ADHD), ASD, mania or BD. Diagnosis of both, cases with CD and TD controls underwent the same clinical assessment. Lifetime diagnosis of internalizing disorders (i.e., depression or disruptive mood dysregulation disorder, anxiety, or OCD) was not an exclusion criteria and controlled for in the statistical analysis.

Environmental risk factors were assessed by a structured medical history interview with the parents or primary caregivers. Full details are available in S1 File. For the analysis here, we summarized the prenatal environmental risk factors as follows: maternal smoking and violence exposure during pregnancy were extracted as binary trait (yes; no), respectively. The difficult family situation score (FamScore, range = 0 to 2) based on parental report of the presence of disharmony in the family (yes; no) and feeling of isolated parenting (yes; no) (for details on the two items: see the Medical History Questionnaire in S1 File). The parental education status (EduPar) was used as proxy for socio-economic status of the family. EduPar was defined as the mean of the highest maternal and paternal self-reported school or occupational degree following ISCED [33] criteria: 0 = pre-primary level of education, 1 = primary level of education, 2 = lower secondary level of education, 3 = upper secondary level of education, 4 = post-secondary level of education, 5 = first stage of tertiary education, 6 = second stage of tertiary education (range = 0 to 6). Participants’ current smoking (number of cigarettes per day) and number of traumata experienced was extracted from the K-SADS-PL [29]. For more details see [34].

DNA was purified from EDTA-blood using the Masterpure DNA purification Kit (Epicentre) with immediate freezing at -80°C as described [35]. Genetic population structure data was available for 97 individuals and is based on common genetic variation (SNPs) as assessed by Illumina Genomic Screening Array. The genetic data are not part of this study. Raw data have been quality controlled and processed using *plink v1*.*9* as published previously [36]; in short: Samples with a genotyping rate < 95%; gender-mismatch, DNA contamination or inbreeding coefficient>0.2 were excluded. SNPs were excluded if MAF<0.05; genotyping rate <95% or imputation score r”<0.3. The first four genetic principal components (PC_1-PC_4) were tested for group-differences.

### Genome-wide methylation analysis

Analysis of the methylome was performed on a total of 102 samples by a modified Methyl-Seq method [37] as previously published [38] using HpaII as the methylation-sensitive enzyme followed by direct Illumina Sequencing on a HiSeq 2000. All samples where preprocessed in one batch and randomly distributed across lanes. For details on library preparation, see S1 File. Quality control using cutadapt version 1.9 included trimming of sequencing adapters and bad quality bases. Reads were hg19 aligned using bowtie2 version 2.2.4. A total of 2 271 932 tags were mapped with an average of 10 059 904 reads per sample prior to filtering. A total of 904 845 tags were measured in at least 50% of the samples. Normalization (tpm; tag per million, DESeq2) and outlier analysis (PCA and hierarchical cluster analysis, S1 Fig in S1 File) was done, resulting in the exclusion of three samples. For functional annotation, DNA features (CpG-island, intergenic region, 2kb upstream of transcriptional start, intronic, exonic, 2kb downstream of transcription stop, 3’UTR, 5’UTR, splicing site) were extracted using the UCSC table tool and RefSeq tracks (1 188 302 tags excluded). A total of 37 753 tags spanning common variants (dbSNP147; MAF≥10%) and tags on the mitochondrial genome (N = 7 892) were also excluded. Furthermore, analysis was limited to tags with at least 5 reads in at least 25 samples of the cases and/or controls, respectively.

Finally, 99 samples and 216 102 tags fulfilled inclusion criteria. (Full details see S1 Fig in S1 File).

### Statistical analysis

All statistical analyses have been done in R version 4.0.3 (2020-10-10). Tags with *FDR*-adjusted *P*-values ≤ 0.05 were considered as significant and selected for validation at expression level. In addition and due to the exploratory nature of the study, tags of interest included in the systems analysis were selected if uncorrected *P*-value ≤ 10E-3. Sensitivity analysis (power) was done in G*power [39].

#### Correcting for cellular composition

Currently, no HpaII-MethSeq reference dataset for specific white blood cell types is available. We performed surrogate variable analysis (SVA [40], R package sva) on all tags which overlap with previously reported Illumina probes that are significantly differentially expressed between cell types [41]. With the full model (see below) 32 surrogate variables (SVs) were identified using the algorithm proposed by Buja and Eyuboglu [42]. To avoid overfitting by including too many variables, only the SV significant (N = 1) at nominal t-test *P* < 0.05 between groups was included in the subsequent models as fixed effect.

#### Differential methylation analysis, model selection

To identify differential methylated tags we performed regression analysis as implemented in the DESeq2 package, with the number of reads at a specific locus as dependent variable and group status as contrast of interest, including the fixed effects age, life-time internalizing disorder, medication, hormonal contraception, number of cigarettes per day, and the SV of interest. DNA quality, concentrations, time to purification, number of total reads and recruiting site were comparable across both groups (all *P*>0.1) and thus not included in the model.

In addition, differentially methylated regions were tested using the *bumphunter* package based on log2 transformed normalized reads. Regional clusters (*clusterMaker*) were defined as adjacent tags with a distance < 500 basepairs (*MaxGap*). Significance was estimated using bootstrapping (*nullMethod*) based on 250 iterations (B) over on the full model as defined above.

#### Sensitivity analysis

Since our models are not corrected for ethnicity and SES we performed a sensitivity analysis on the main hit, by comparing the effects of group on methylation between the original “un-corrected” model and respective models corrected for the environmental risk factors including Parental Education (EduPar), or the population structure components PC_1 to PC_4. We also performed a sensitivity analysis for EduPar and of internalizing disorder history on the overall EWAS dataset comparing the effects of both models.

#### Enrichment analysis

We tested the null-hypothesis of “no association of a tag with a given annotated feature” implementing Bonferroni corrected Fisher’s exact test comparing tag status (*P* ≤ 10E-3 yes-no) versus feature status (yes-no). We tested the list of genes adjacent to hyper- or hypomethylated tags and genes adjacent to differentially methylated loci. For GO-term enrichment analysis we adapted the gprofiler2 [43] package with *correction_method* =“g_SCS” to account for multiple testing as well as the diacyclic graph structure of the ontologies. Analyses were compared against all genes detectable in our analysis (reference gene-universe). We tested the list of genes within +/-2kb of the tag or region of interest, respectively.

#### Enrichment in gene-expression modules co-regulated during brain development

We tested our genes of interest (i.e., +/-2kb) proximal to significant (*P* ≤ 10E-3) tags or regions for enrichment within the previously published 29 gene network modules [44] co-regulated during human brain development using Bonferroni corrected Fisher’s exact test (29 modules, 2 gene-lists; http://www.brain-map.org).

#### Path-analysis

Was performed using the *lavaan* and the *semPlot* [45] packages in R implementing the diagonally weighted least squares (DWLS) estimators and a non-linear minimization subject to box constraints (NLMINB) optimizer with CD group status as main outcome variable. The environmental risk factors included were i) maternal smoking during pregnancy, ii) maternal exposure to violence, iii) the adverse parenting risk score (FamScore), iv) parental education (EduPar), and v) number of traumata experienced by the participant. The respective epigenetic signature were defined as i) the log2 reads of the top hit identified (Epi_top_hit) or ii) the Eigenvalue of all tags with a nominal significance of *P* ≤ 10E-3 (Epi_all); or iii) the subgroups there of based on brain expressed genes (Epi_M_all), or only on the associated brain developmental modules M2 (iv) (Epi_M2) and M15 (v) (Epi_M15), respectively. As covariates of no interest, we included age at recruitment, contraceptives, cigarettes per day, medication, history of internalizing disorder and the surrogate variable for cellular composition. We tested i) the direct effects on CD status of the five environmental factors and the respective epigenetic signature correcting for the covariates, ii) the effect of each risk factor on the respective epigenetic signature, correcting for covariates and iii) the mediation effects of the environmental risk factors via the Epigenetic factors. All variables have been loaded in parallel. For direct comparison, we also optimized a model without direct effects only (corrected for all covariates). In total, 5 mediation models have been optimized, each with 27 free parameters. Uncorrected p-values are reported. Correction for multiple testing was not considered, as epigenetic signatures and paths within the models are not independent.

### Functional analyses of SLITRK5 methylation

For 21 of the 37 individuals of the Frankfurt site (14 TD controls, 7 CD cases) we were able to establish lymphoblastic cell lines, as published previously [46]. (Only participants from Frankfurt agreed to have cell lines generated within the written informed constent). A total of 1x10E6 cells were harvested in exponential log phase and immediately subjected to RNA extraction using the NucleoSpin RNA Kit (Macherey and Nagel) according to the manufacturer’s protocol. The Revert Aid H- first strand cDNA Synthesis Kit (Thermo Fisher Scientific) using random hexamers was applied to generate cDNA. Real-time PCR using Prima quant qROX (Steinbrenner) and the Universal Probe Library (Roche) was performed in technical triplicates on a StepOne Plus Cycler using the recommended settings. Geometric mean of GUSB and GADH expression was used as reference. Primers for the reference and the two target genes *SLITRK5* and *MIR4500HG* were designed with the Universal Probe Library Assay Design Center. Relative Quantification was performed using the -ΔΔCt method [47] with the mean ΔCt of all samples as reference.

## Results

### Sample description

Overall, 49 cases with CD and 50 TD controls matched for age and pubertal status passed quality check (Table 1). Population structure component PC2 was significantly different between the groups (*P* = 0.019). Significantly (*P* ≤ 0.05) more cigarettes per day were smoked in the CD group compared to TD controls. Furthermore, cases showed a higher frequency of a lifetime diagnosis of internalizing disorders and medication. Females with CD compared to TD controls showed an increased exposure to violence during pregnancy, and adverse parenting conditions (FamScore) but not maternal smoking. Parental education (EduPar), on average, was significantly lower in the CD group (Table 1). No differences in reads between groups were detected (*P* > 0.1). The available sample size (n = 99) and the acceptance of a beta error > = 0.2 and an alpha error < = 10E-3 in a linear regression model with 1 variable tested (case/control) and 6 covariates as fixed effects of no interest, allows detecting small to medium effects of f^2^ > 0.18.

### Differential methylation analysis

Here, HpaII-reads reflect un-methylated tags, thus, a decrease in reads (i.e., a negative effect size) reflects an increase in methylation. Descriptively, 237 tags were associated with group status at uncorrected *P* ≤ 10E-3 (S1 Table). Of these, 108 tags were less methylated (log2FC > 0) in cases whereas 129 tags showed an increased methylation. When correcting for EduPar the effects of the 237 tags of interest were comparable (Spearman’s rho 0.946).

**Fig 1:**
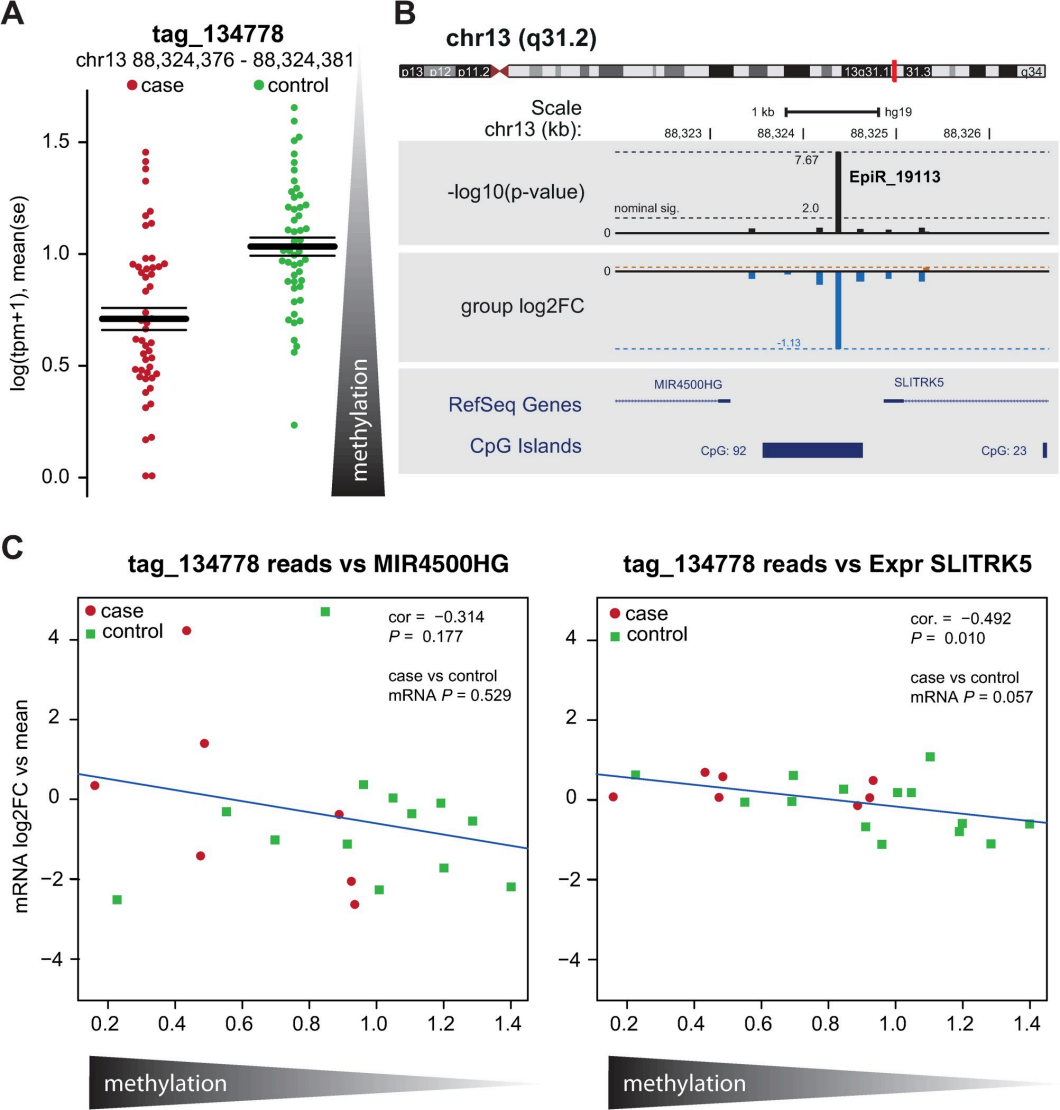
Differentially methylated loci. **A)** Hypermethylation of tag 134778 in 49 cases versus 50 controls. (log2FC -1.13 SE: 0.20 *FDR* = 0.0046). Log transformed number of unmethylted tags per million reads (log (tpm +1)) measures the number of un-methylated CCGG CpG regions. Thus, decreased log(tpm+1) counts indicate an increase in methylation in cases. **B)** tag_134778 maps to an intergenic region on chr 13 q31.3 upstream of the miRNA gene 4500HG (- coded) and the *SLITRK5* gene (+ coded) within the CpG island 92. log2FC values and corresponding–log10 (uncorr *P*-values) are shown for the adjacent region. **C)** mRNA expression levels of the two adjacent genes (log2FC against mean expression) correlated against the observed normalized and log transformed read counts of the *SLITRK5* methylation site for 21 available lymphoblastoid cell lines. cor.: Spearman correlation; case vs control comparison Wilcoxon-test.

The tag with the strongest association (S1 Table) is located at chr13q31.2 within a CpG island and a glucocorticoid receptor binding site upstream of the neuronal expressed *SLITRK5* and downstream of the miRNA *MIR4500HG*. Tag_134778 showed a significantly decreased read count (i.e., increased methylation) in cases (log2FC -1.13, SE: 0.20, *FDR* = 0.0046; Fig 1A and 1B). This effect was still significant (p-value < 5*10E-5) when controlling for population structure, site, environmental risk factors or when not controlling for internalizing disorder history (S2 Fig in S1 File). At mRNA level *in vitro*, we observe an expression of *SLITRK5* which is significantly correlated (corr = -0.492, *P*-value = 0.010) with normalized read counts (Fig 1C). We observe a trend that on average expression is higher in cases compared to controls (*P*-value = 0.057). Thus, the increased methylation (i.e., reduced reads) increased the expression of *SLITRK5*. We did not observe a significant correlation or group difference of the expression of the downstream located *MIR4500HG* (all *P* > 0.05, Fig 1C) [51].

None of the 8,108 tested regions was significant at *FDR* ≤ 0.05. A total of 12 regions were significantly associated with CD at a nominal *P*-value < 10E-3, among them the promoter region of *SLITRK5* (log2FC = -1.187, nominal *P*-value = 8x10E-4).

### Gene list enrichment analysis

**Fig 2:**
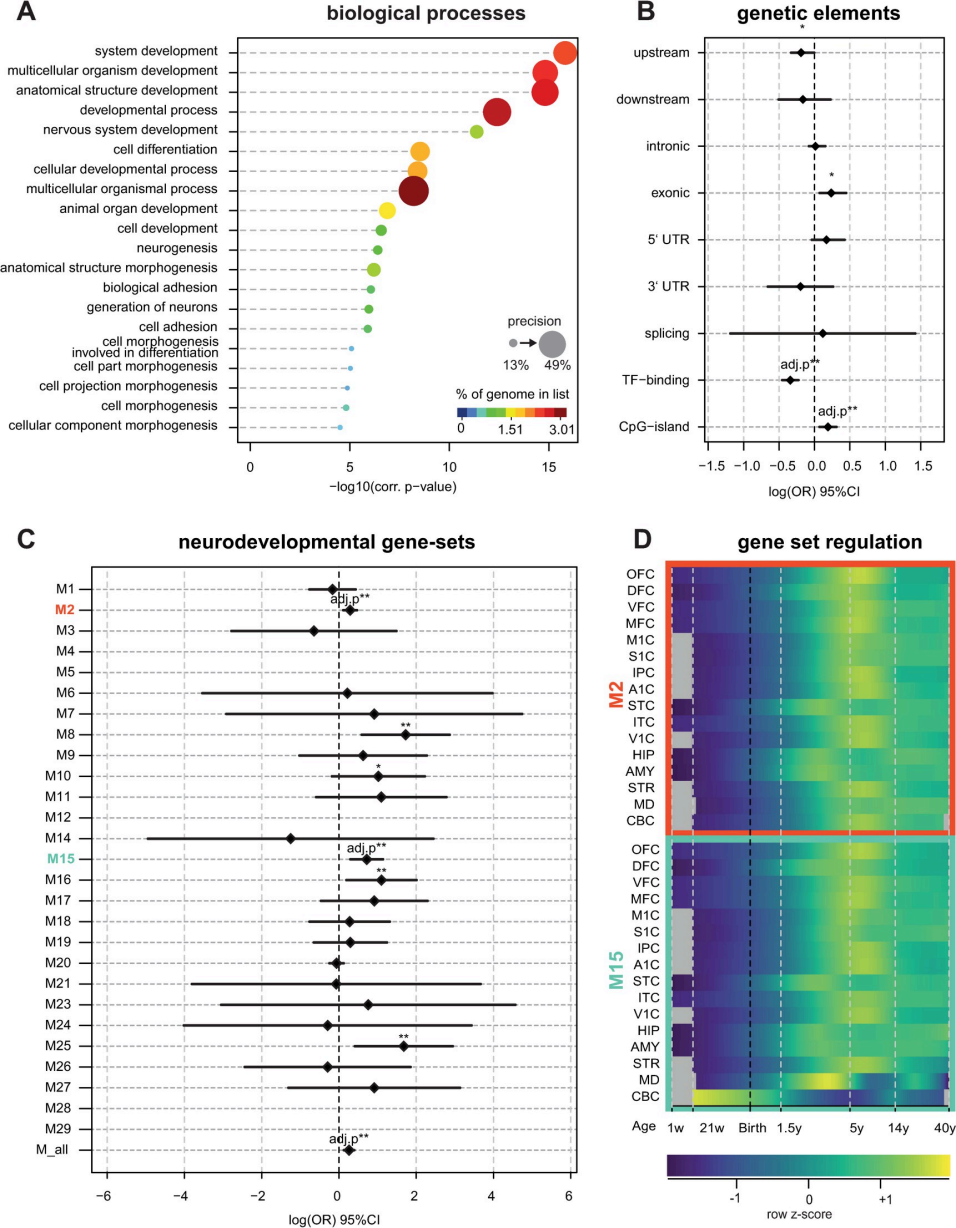
Enrichment analyses. **A)** GO-term enrichment analysis: Differentially methylated regions were mapped to respective RefSeq annotated genes using the gprofiler2 algorithm accounting for the topology of GO-terms. Top 15 significant findings for GO-biological processes are shown, for full table of results see S2 Table. Correction for multiple testing is inherent to the used algorithm. **B)** Enrichment tests of differentially methylated loci within annotated functional genomic regions. **C)** Enrichment of adjacent genes among developmental brain transcriptome networks: Kang et al (2010) [44] have identified 29 distinct co-regulated gene networks that are activated during brain development and ageing. We tested the list of differentially methylated genes for enrichment in any of the 29 modules using Fisher-exact test correcting for multiple testing and identified significant overlap with modules 2 and 15 as well as among genes expressed during brain development. **D**) Normalized Eigenvalue plots of the two modules enriched for tags across time and brain regions. Annotated function is as published in the original paper^41^. Module M2 is activated during the late prenatal brain development in cortical areas and specifically in the amygdala and hippocampus. During later development the other brain areas follow. After puberty, the brain modules are constant. M15 is downregulated in the cerebellum during development and perinatally activated in the medio dorsal nucleus of the thalamus, hippocampus, amygdala, and striatum until early childhood. M15 gets activated in the other brain areas during late childhood, early puberty. Abbreviations: OFC: Orbital Prefrontal Cortex; DFC: Dorsolateral Prefrontal Cortex; VFC: Ventrolateral Prefrontal Cortex; MFC: Medial Prefrontal Cortex; M1C: Primary Motor Cortex; S1C: Primary Somatosensory Cortex; IPC: Posterior Inferior Parietal Cortex; A1C: Primary Auditory Cortex, STC: Superior Temporal Cortex; ITC: Inferior Temporal Cortex; V1C: Primary Visual Cortex; HIP: Hippocampus; AMY: Amygdala; STR: Striatum; MD: Mediodorsal Nucleus of the Thalamus; CBC: Cerebellar Cortex.

The list of genes adjacent to differentially methylated loci was significantly (corrected *P*-value<10E-3) enriched for neurodevelopmental processes including *nervous system development*; *neurogenesis*; *neurite outgrowth and development* (Fig 2A). At cellular compartment level, significant enrichment for *cell junction*, *glutamatergic synapse* and *neuron-to-neuron synapse* was identified. The genes within differentially methylated regions were enriched for *system development*. In addition, significant enrichment of differentially methylated loci was observed in CpG islands (Bonferroni *P* ≤ 0.01, Fig 2B).

Comparing the previously published gene modules [44] activated during neuronal development to the here identified list of differentially methylated genes we observed a significant (adj. *P*-value < 0.01) enrichment in the overall brain expressed genes (Fig 2C and 2D), as well as in two specific gene networks, namely M2 and M15. Module M2’s expression is progressively increasing starting at the late embryonic period and plateauing during puberty [44]. Similarly, module M15 overall increases during development and specifically prenatally and in early infancy in neocortical, hippocampal, amygdaloidal and striatal regions. M15 is also highly upregulated prenatally in the cerebellum [44].

### Mediator analysis

In the model without any mediation effects, including all environmental risk factors and covariates, only parental education was significantly associated with CD (-0.435, *P* = 0.010). In all the mediator models, the differentially methylated epigenetic signatures (Epi_top_hit, Epi_all, Epi_Mall, Epi_M2, Epi_M15) where—as expected—directly correlated with CD risk in all five models (all |beta| > 1.01, *P* < 0.001, all models converged with *P* < 0.001).

Within the mediation models (S3 Table), parental education correlated with epigenetic signatures of Epi_M15 (differentially methylated tags of module M15) but not with our top Hit or the other epigenetic signatures tested. In this model the direct effect of parental education was not significant (P > 0.05). However, the indirect effect via the overall epigenetic signature was (Epi_M15 x EduPar beta = -0,261, *P* = 0.027). In summary, our finding suggests that CD is associated with a gene set specific epigenetic signature related to parental education.

## Discussion

This is the first study to describe genome-wide epigenetic methylation patterns in female adolescents with CD, studying their role at the systems level, and investigating the mediation of environmental risk by DNA methylation.

Similar to genetic studies in CD with mixed gender cohorts [48] or in epigenetic studies on stress or aggression in young people, single gene methylation patterns did not strongly differ between females with CD and TD.

However, a significant difference was observed for *SLITRK5* hyper-methylation, which was further associated with an increased expression of the gene in females with CD-cases. The positive correlation between methylation and expression possibly underlies blocking of an inhibitory interaction. Indeed, the identified CpG is located within a glucocorticoid receptor (GR) and Pol-II transcription factor binding site. The GR-mediated transcriptional response is highly dynamic and interacts with inhibitory transcription factors (for a review see [49]). Thus, we propose a model in which increased methylation inhibits accessibility of the GR and inhibitory co-factors leading to an increased expression. This, however, remains to be validated in neuronal cells.

The synaptic adhesion protein SLITRK5 supports trafficking of the BDNF-dependent neurotrophin receptor [50] and it is ubiquitously expressed in the human developing brain as well as in the adult occipital and frontal lobes [51]. Functionally, it is associated with neurite development and synapse assembly, specifically with the development of excitatory and inhibitory synapses on midbrain dopaminergic (mDA) neurons [52]. Previously *SLITRK5* deleterious mutations have been associated with Obsessive Compulsive Disorder [53]. In mice, knock-out induced excessive grooming as well as anxiety-like behavior and reduced striatal volume [52]. Interestingly, overexpression of Slitrk5 in mice led to a reduction in the complexity of dendritic arborization in dopaminergic neurons. Similar pathomechanisms, i.e., a deregulation of excitatory and inhibitory synapses on mDA neurons might underlie the etiology of CD.

Further exploration of differentially (uncorr. *P-*value < 10E-3) methylated tags at systems level showed a significant (corr. *P-* value < 0.05) enrichment within CpG sites. Enrichment analysis of the adjacent genes clearly pointed to neurodevelopmental and neuronal pathways. Specifically, we identified several genes associated with synaptic transmission (e.g., glutamate receptors *GRIK3*, *GRIN2A*, or *GRM3*, serotonin receptor subunits *HTR2C*, *HTRA3*, or dopamine receptor *DRD1*) as well as neuronal adhesion (e.g., contactin genes *CNTN3* and *4*) and synapse development (e.g., *HOMER1*, *DLGAP1*). The identified enrichment for differential methylation in genes active during prenatal neuronal development led us to the hypothesis that the etiology of females with CD is of developmental origin. Based on the observation that the Eigenvalue of Module M2-reads negatively correlated with CD (i.e., higher methylation in CD-cases) in our late/post-pubertal sample suggests that the epigenetic regulation of the prenatally activated neurodevelopmental genes is too strong. Similarly, the genes of the Module M15, which are active in the limbic and neocortical system showed an increased epigenetic regulation. We thus suggest that the epigenetic signature of female adolescent CD affects the development of structures underlying cognition, affective states and emotion regulation [54]. This corroborates meta-analytic findings of a reduced limbic activation in adolescents with disruptive behavior disorders and conduct problems [55].

We further explored the hypothesis that the long-term influence of environmental risk on psychopathology is mediated by epigenetic alterations of these co-regulated brain developmental gene modules. Overall, we observed a significant positive correlation of parental education with CD status, which is mediated by epigenetic signatures. Descriptively, increased education was associated with an increased Epi_M15 signature, which itself negatively correlated with an increased risk for CD. Please note, in our experimental setup an increased read reflects a decreased methylation. Thus, increased methylation of module 15 genes (Epi_M_15) is associated with CD risk and potentially under the influence of parental education. However, due to the lack of longitudinal data, we cannot conclusively state, if the signatures are a consequence of parental education or of CD.

Parental education can be a proxy for childhood socio-economic status (SES) and low SES during infancy has previously been associated with global hypo-methylation in adults [56]. Correlation between SES and methylation of genes coding for the oxytocin receptor, vasopressin and *FKBP5* have been reported [57]. Furthermore, childhood SES has been associated with altered methylation of signaling pathways including the BDNF-receptor and the MAPK pathways [58]. Our findings corroborate this association by reporting a nominal differential methylation (*P* < 10E-3) in females with CD of the BDNF and MAPK associated genes *PTEN*, *NTRK1* or *NTRK3* (neurotrophic receptors), and extends these studies by mapping the protective effect of high SES [8, 59] to a differentially methylated signature of gene networks related to the emotion regulation system.

Thus, we hypothesize that the high parental education as a proxy for SES may counterbalance epigenetic patterns of females with CD.

A major limitation of this study is that no HpaII-Meth-Seq reference for cellular composition is available. In our approach, one surrogate variable showed nominally significant differences between groups and were included in our models. To identify SVs we implemented two algorithms. However, the algorithm proposed by Leek [59] suggests that no significant surrogate variable is underlying the data. Thus, it is unlikely that differences in cellular composition, or more specifically hidden factors, are biasing our results.

Furthermore, due to power constraints we did not include an environmental factor based differential methylation analysis or diagnosis by risk factor interaction analysis but rather explored the associations in a SEM model. We are aware that environmental risk factors such as SES do have an overall effect on methylation [56]. However, we also showed that the effects of the differentially methylation loci in our cohort are stable when corrected for SES. At technical level, HpaII methyl sequencing (in contrast to MspI/HpaII) does not allow comparison between the individual loci or to calculate M-values comparable to Illumina Data, since normalization for restriction enzyme efficacy is not possible.

Tags included in the downstream analyses were selected based on uncorrected levels of significance. We thus expect an increased chance for false positive and negative findings. While functional analysis of *SLITRK5* and the *FDR* < 0.05 indicates a true positive finding, tags of interest included in the post-hoc path and mapping analysis might be biased and should thus only be considered within the system-based analysis. Here, larger sample sizes are needed, specifically to replicate the systems-based and path-analysis of this study.

In this study here, we decided to control for lifetime internalizing disorders with the aim to avoid identifying methylation signatures associated with general psychopathology. This approach may reduce thee generalizability to CD without comorbidity only. However, our main effect was not altered by omitting the correction for internalizing disorders.

In addition, given the limitations of the path-analysis here, the findings from the structural equation modelling need to be interpreted with caution: First, sample size is low, compared to the number of paths modelled, and thus, the models are prone to overfitting. The reported effects need to be validated in an independent cohort. Second, cross sectional data only allows identification of correlations. Although most of the tested environmental factors are antecedents of the epigenetic markers, we cannot fully conclude that the epigenetic signatures themselves are causal for CD. Finally, the study focused on females only, and thus lacks a comparison to male individuals.

### Conclusion

The results of our exploratory system wide approach allow the conclusion that biological and environmental risk for CD in females is associated with complex epigenetic changes underlying differentially orchestrated gene networks. One of the candidates identified here is the neurite regulator *SLITRK5*, which lets us suggest that its increased expression mediated by an increased methylation is related to CD diagnosis in the female cohort. Although explorative in nature, this work highlights that SES is a protective factors mediated by differential epigenetic markers in females with CD. These are likely to regulate genes associated with neuronal function and development of CD associated brain and neurotransmission systems. To conclude if these changes ultimately result in the CD phenotype we suggest longitudinal studies.

## Supporting information

S1 Table Linear model results. (XLSX)

S2 Table GO term enrichment results. (XLSX)

S3 Table Results path analysis. (XLSX)

S1 File (DOCX)
