# Supplementary material for: The methylome in females with adolescent Conduct Disorder: Neural pathomechanisms and environmental risk factors
Source: PLoS One. 2022 Jan 28;17(1):e0261691. doi: 10.1371/journal.pone.0261691 (PMC8797262; doi:10.1371/journal.pone.0261691)
Supplement: S1 File — (DOCX) [file pone.0261691.s004.docx]

S1 File

The Methylome in Female Adolescent Conduct Disorder: Neural Pathomechanisms and Environmental Risk Factors

AG Chiocchetti^1^, A Yousaf^1^, R Waltes^1^, A Bernhard^1^, A Martinelli^1^, K Ackermann^1^, D Haslinger^1^, B Rotter^2^, N Krezdorn^2^, K Konrad^3,4^, G Kohls^3^, A Vetro^5^, A Hervas^6^, A Fernández-Rivas^7^, CM Freitag^1^

1 University Hospital Frankfurt; Department of Child and Adolescent Psychiatry, Psychosomatics and Psychotherapy; Frankfurt am Main, Germany

2 GenXPro GmbH, Frankfurt am Main, Germany

3 Child Neuropsychology Section, University Hospital, RWTH Aachen, Department of Child and Adolescent Psychiatry, Psychosomatics and Psychotherapy, Aachen, Germany

4 JARA BRAIN Institute II, Molecular Neuroscience and Neuroimaing, Institute of Neuroscience and Medicine (INM-11), Research Center Juelich

5 Szeged University, Department of Pediatrics and Pediatrics health center, Child and Adolescent Psychiatry, Szeged, Hungary

6 Hospital Universitario Mutua de Terrassa, Child and Adolescent Mental Health Service, Barcelona, Spain

7 Basurto University Hospital, Psychiatric Service, Osakidetza, Bilbao, Spain

**Content**

[1 Supplementary Methods 2](#_Toc61690242)

[1.1 Assessment instruments 2](#_Toc61690243)

[1.2 Matching procedure 5](#_Toc61690244)

[1.3 Methylation Sequencing 5](#_Toc61690245)

[2 Supplementary Tables 7](#_Toc61690246)

[3 Supplementary Figures 7](#_Toc61690247)

[4 References 8](#_Toc61690248)

# Supplementary Method

## Assessment instruments

K-SADS-PL: Current and history of psychiatric disorders (affective, psychotic, anxiety, behavioral, substance abuse, and other psychiatric disorders) were assessed by separate semi-structured diagnostic interviews using The Schedule for Affective Disorders and Schizophrenia for school-age children - Present and Lifetime version (K-SADS-PL) with participants and parents and / or caretaker conducted by trained masters- and doctoral-level staff. Interrater reliability (Cohen’s κ = .91) and rater agreement (95%) of current CD were high. Also, for current and past ADHD,, oppositional defiant-, depressive and posttraumatic stress disorder interrater reliabilities (Cohen’s κ = .50 - .95) and rater agreements (92 - 95%) were moderate to high[1].

IQ: IQ was estimated from the matrix reasoning and vocabulary subtests of the Wechsler Intelligence Test (11 - 16 years: 58; > 16 years: 59) or the Wechsler Abbreviated Scale of Intelligence [2–4].

PDS: Pubertal status was assessed via self-report using the Pubertal Development Scale[5] asking about pubertal growth (e.g. changes in body hair, voice or breast development) with four response options (not yet started, barely started, definitely started, seems complete) resulting in a 5-level categorical scale (0 = pre-pubertal, 1 = early-pubertal, 2 = mid-pubertal, 3 = late-pubertal, 4 = post-pubertal).

Semi-structured psychosocial and medical history questionnaire (Full version see below): Psychosocial and medical risk factors were assessed using the Medical History, a semi-structured clinical interview conducted by trained masters- and doctoral-level staff designed to assess current and past environmental influences and risk factors of children and adolescents. Parents and/or caregivers were interviewed regarding 1. pregnancy and birth history, 2. specific psychosocial risk factors during the early development, 3. developmental milestones, 4. nursery and kindergarten, 5. school career, 6. chronic medical problems, 7. parental education status, 8. information about the family, such as single parenting, and 9. psychiatric disorders in the family. The full interview consists of ~60 binary (yes/no) or categorical (e.g. employment) questions. The full questionnaire can be found below.

**Semi-structured psychosocial and medical history questionnaire**

Please note that the published questionnaire is for documentation purposes only and not to be used by other studies. If any readers are interested in doing so on a collaborative basis, please contact the femNAT-CD consortium ([christinemargarete.Freitag@kgu.de](mailto:christinemargarete.Freitag@kgu.de)).

## Matching procedure

To account for potential modifier effects at the epigenetic level we compared the CD and control samples for age at blood drawing (BD), pubertal status, hormonal contraception, cigarettes per day, days between BD and purification as well as the 260/280 ratio implementing the *matchIt* package in R (method=“nearest”). Post-matching tests showed no differences (p-value > 0.15) between cases and controls with respect to age, pubertal status, days to purification or 260/280 ratio. Hormonal contraception (p-value = 0.025) or cigarettes smoked per day (p-value < 0.001) could not be matched and were thus adjusted for in statistical analysis.

## Methylation Sequencing

Methyl-Seq: Genome-wide analysis of CpG DNA methylation was performed by a modified Methyl-Seq method [7] as previously published [8]. In brief, HpaII was used as the methylation-sensitive enzyme, recognizing non-CpG-methylated CCGG sites. After digestion with HpaII, “TrueQuant” Y-adapters (GenXPro) suitable for direct Illumina Sequencing were ligated to the fraction of molecules between 100 and 500 bps, which was prior separated from larger DNA fragments using AMpureBeads (Beckman-Coulter [A63882]). Subsequently, the larger DNA was randomly sheared using a bioruptor (Diagenode) to a size of 300–500 bps, and adapters containing Illumina P7 priming sites were ligated. The products were PCR amplified using P5 and P7 primers (Illumina) with 10 cycles and sequenced on an Illumina Hiseq4000 machine with 76 cycles. Quality reads starting with the HpaII-specific recognition sequence (CCGG) were quality-trimmed and mapped to the human genome (hg19) using bowtie2 [9]. For each genomic HpaII site, reads starting at that position were quantified in each library.

Genomic annotation: The RefSeq track as well as the CpG island track from the UCSC table browser (https://genome.ucsc.edu/cgi-bin/hgTables) were used to annotate genomic restriction sites. Regions up to 2 kb upstream of the transcription start site (TSS) of genes contained in the RefSeq track were defined as promoter regions. Accordingly, downstream regions were defined as genomic regions up to 2 kb downstream of the 3' UTR. In order to annotate miRNA promoter regions, miRNA TSS information was retrieved from the miRStart web service (http://mirstart.mbc.nctu.edu.tw/).

Tag selection: HpaII restriction sites can be compromised by overlapping SNPs. These SNPs can either induce technical artifacts or may directly infer changes in methylation. However, we are currently unable to account for these variants in our analysis and thus decided to exclude any epigenetic tag with the restriction site carried a potential common variant with a MAF≤10%. SNPs and MAFs were based on the dbSNP147 hg19 annotated dataset. Finally 259 742 tags were included in downstream analyses.

Primary quality control (QC): Raw reads were normalized to the overall number of reads per sample (tags per million; tpm), log(tpm+1) transformed and plotted for each sample to visualize normalization. Distribution and outliers were comparable across all samples and no batch effects were detected. Samples were clustered using the Euclidean distance and “Ward.2D hierarchical cluster algorithm(Supplementary figure 1). In addition PCA and MDS based inspection of samples led to exclusion of two outliers.

All Preprocessing, QC, MethSeq analysis and the respective source code are available on github <https://achiocch.github.io/femNATCD_MethSeq/>

Raw data files used for the analysis are available under

<https://osf.io/erqwp/?view_only=58bd1878c6df4c178d071c7803fdf545>

# Supplementary Tables

All supplementary tables are provided as excel sheets

Supplementary Table S1: Linear model results

Supplementary Table S2: GO term enrichment results

Supplementary Table S3: Results path analysis.

# Supplementary Figures


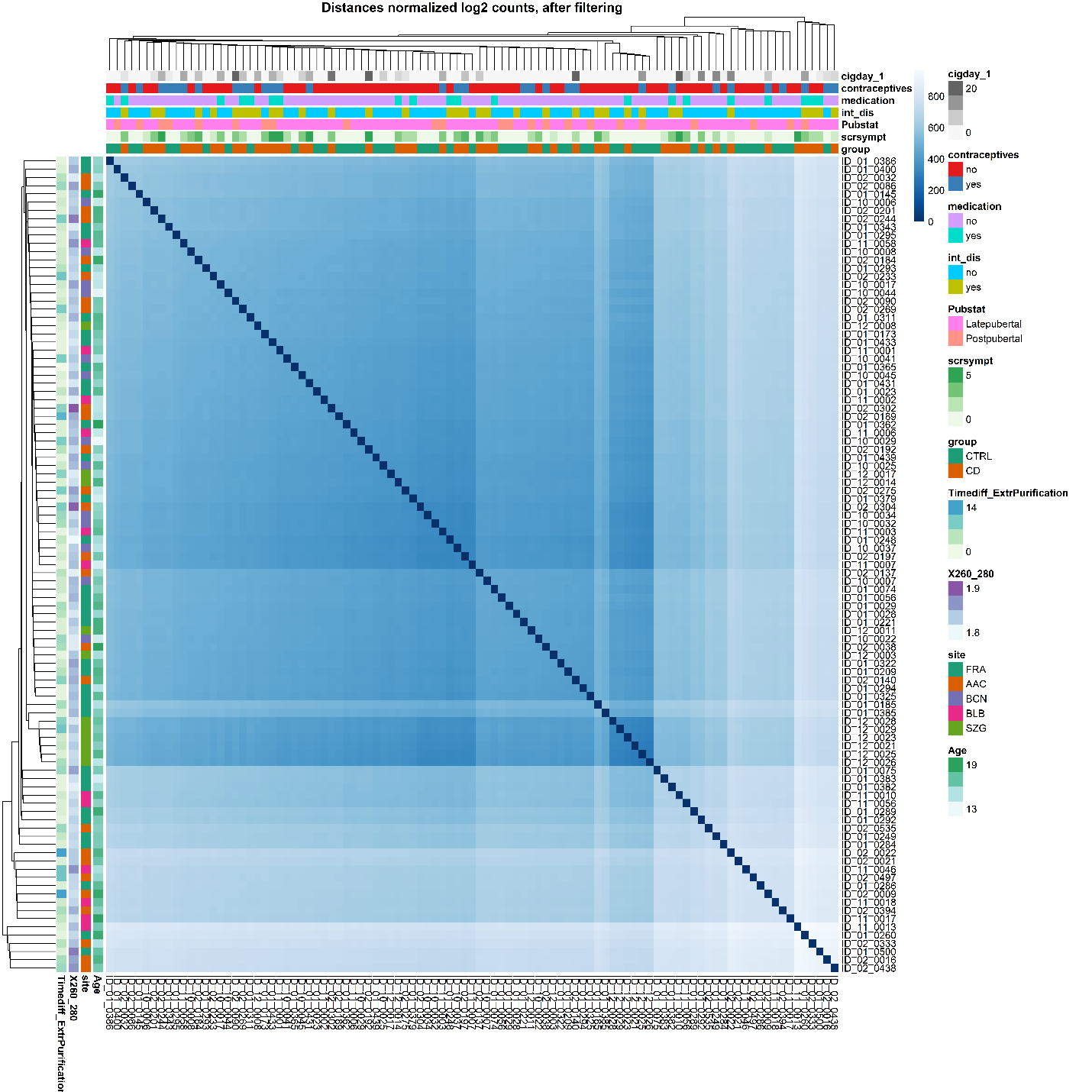


**Supplementary Figure 1 Cluster based Quality control:** Heatmap of sample distance based on log(tpm+1) shows no obvious case control stratification. One site specific cluster can be identified, with no enrichment for cases or controls. Abbreviations: scrsympt: CD symptom count [CBCL], cigday_1: cigarettes per day, int_dis: internalizing disorder life-time diagnosis, Pubstat: Pubertal status, Timediff_ExtrPurification: days between blood sample collection and DNA extraction. X260_280: DNA quality absorbition ratio 260nm/280nm; sites: FRA: Frankfurt, AAC Aachen, BCN Barcelona, BLB Bilbao, SZG Szeged.


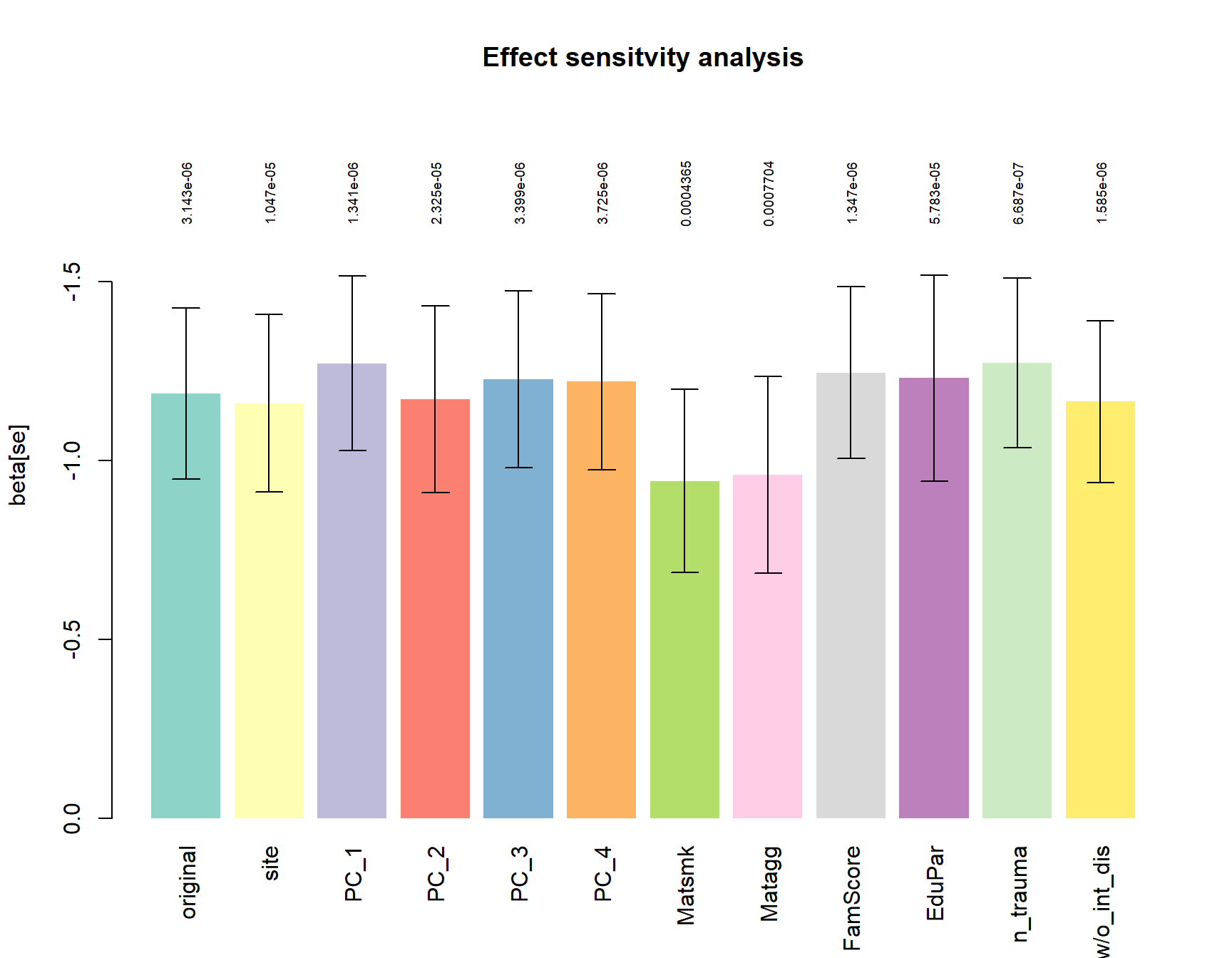


**Supplementary Figure 2: Sensitivity analysis of the top Hit.** The group effect of functionally validated tag (top Hit) proximal to the SLITRK5 gene was tested for stability when correcting for site, genetic population structure (PC_1 to PC_4), Maternal smoking during pregnancy (MatSmk), Maternal exposure to aggression during pregnancy (Mat Agg), Difficult Family situation (FamScore), Parental Education (EduPar) and number of trauma experienced by the proband. Effect sizes and standard deviation are plotted (inversely) with the respective p-value. In all models, the effect remained significant.

References

**1**. Kaufman J, Birmaher B, Brent D, Rao U, Flynn C, Moreci P, et al. Schedule for Affective Disorders and Schizophrenia for School-Age Children-Present and Lifetime Version (K-SADS-PL). Initial reliability and validity data. J Am Acad Child Adolesc Psychiatry. 1997; 36:980–8. doi: 10.1097/00004583-199707000-00021 PMID: 9204677.

**2**. Wechsler D. Wechsler Abbreviated Scale of Intelligence. San Antonio: TX: Psychological Corporation; 1999.

**3**. Wechsler D. Wechsler Intelligence Scale for Children—Fourth Edition (WISC-IV). San Antonio: TX: Psychological Corporation; 2003.

**4**. Wechsler D. Wechsler adult intelligence scale–Fourth Edition (WAIS–IV). San Antonio: TX: NCS Pearson 22; 2008.

**5**. Petersen AC, Crockett L, Richards M, Boxer A. A self-report measure of pubertal status. Reliability, validity, and initial norms. J Youth Adolesc. 1988; 17:117–33. doi: 10.1007/BF01537962 PMID: 24277579.

**6**. Organisation for Economic Co-operation and Development. Classifying educational programmes: Manual for ISCED-97 implementation in OECD countries. Paris: OSCED; 1999.

**7**. Brunner AL, Johnson DS, Kim SW, Valouev A, Reddy TE, Neff NF, et al. Distinct DNA methylation patterns characterize differentiated human embryonic stem cells and developing human fetal liver. Genome research. 2009; 19:1044–56.

**8**. Zawada AM, Schneider JS, Michel AI, Rogacev KS, Hummel B, Krezdorn N, et al. DNA methylation profiling reveals differences in the 3 human monocyte subsets and identifies uremia to induce DNA methylation changes during differentiation. Epigenetics. 2016; 11:259–72.

**9**. Langmead B, Salzberg SL. Fast gapped-read alignment with Bowtie 2. Nat Meth. 2012; 9:357–9. doi: 10.1038/nmeth.1923 PMID: 22388286.
